# Sleep deprivation and stress: a reciprocal relationship

**DOI:** 10.1098/rsfs.2019.0092

**Published:** 2020-04-17

**Authors:** Mathieu Nollet, William Wisden, Nicholas P. Franks

**Affiliations:** 1 Department of Life Sciences, Imperial College London, London, UK; 2 UK Dementia Research Institute at Imperial College London, London, UK; 3 Centre for Neurotechnology, Imperial College London, London, UK

**Keywords:** glucocorticoids, non-rapid eye movement sleep, rapid eye movement sleep

## Abstract

Sleep is highly conserved across evolution, suggesting vital biological functions that are yet to be fully understood. Animals and humans experiencing partial sleep restriction usually exhibit detrimental physiological responses, while total and prolonged sleep loss could lead to death. The perturbation of sleep homeostasis is usually accompanied by an increase in hypothalamic–pituitary–adrenal (HPA) axis activity, leading to a rise in circulating levels of stress hormones (e.g. cortisol in humans, corticosterone in rodents). Such hormones follow a circadian release pattern under undisturbed conditions and participate in the regulation of sleep. The investigation of the consequences of sleep deprivation, from molecular changes to behavioural alterations, has been used to study the fundamental functions of sleep. However, the reciprocal relationship between sleep and the activity of the HPA axis is problematic when investigating sleep using traditional sleep-deprivation protocols that can induce stress *per se*. This is especially true in studies using rodents in which sleep deprivation is achieved by exogenous, and potentially stressful, sensory–motor stimulations that can undoubtedly confuse their conclusions. While more research is needed to explore the mechanisms underlying sleep loss and health, avoiding stress as a confounding factor in sleep-deprivation studies is therefore crucial. This review examines the evidence of the intricate links between sleep and stress in the context of experimental sleep deprivation, and proposes a more sophisticated research framework for sleep-deprivation procedures that could benefit from recent progress in biotechnological tools for precise neuromodulation, such as chemogenetics and optogenetics, as well as improved automated real-time sleep-scoring algorithms.

## 1. What is sleep?

Sleep in mammals, as defined by its behavioural and physiological features, includes two distinct global activity states: rapid eye movement (REM) sleep and non-REM (NREM) sleep. These two states are typically characterized by electrophysiological measures including electroencephalography (EEG) and electromyography (EMG), as well as electrooculography in humans. NREM sleep, which is further divided into four substages of increasing depth in humans, is characterized by high-amplitude, low-frequency EEG oscillations and behavioural quiescence with a relaxed muscle tone. Apart from the typical ocular movements, REM sleep (or paradoxical sleep) is characterized by low-amplitude, high-frequency EEG oscillation in association with muscle atonia. NREM and REM sleep alternate in cycles, with NREM sleep preceding REM sleep episodes. In the mammalian brain, the sleep–wake cycle is orchestrated by a complex network of discrete neuronal populations that induce sleep or wakefulness via promoting or suppressing effects [1]. Nevertheless, some aspects of sleep homeostasis are different between humans and rodents. Human sleep is monophasic and typically consists of a single block of three to five cycles of sleep stages, usually happening during the night [1]. Despite variations in the daily duration of sleep and temporal pattern of vigilance states across laboratory rodents [2], sleep in these animals is generally polyphasic with repeated sleep episodes throughout the 24 h light–dark cycle [1]. In addition, commonly used laboratory rodents are nocturnal and preponderantly sleep during the light period [1].

## 2. Why do we need to sleep?

If the functions of sleep remain blurry, it has been proposed to play a key role in optimizing the conservation and utilization of energy by reallocating energy reserves to essential biological processes such as cellular maintenance, anabolism, immune function and neural plasticity rather than wake-related features such as vigilance, foraging and reproduction [3]. Furthermore, by overcoming the energy deficits accumulated during wakefulness and by preparing the organism for the next wake-related energy expenditure, sleep may further support fundamental mechanisms such as brain waste clearance via the glymphatic system [4] or daily stress resistance [5]. The disconnection from the environment during sleep has also been proposed to be critical for the homeostatic plasticity of the brain. The synaptic homeostasis hypothesis suggests that the potentiation of synaptic connections throughout the brain that happens during wakefulness, supporting learning functions but also increasing the demand for energy and cellular supplies, is then normalized during sleep that can restore cellular homeostasis [6]. This re-normalization of synaptic strength could favour memory acquisition, consolidation and integration during sleep [6]. These interrelated phenomena have been described as vital for the organism, both ontogenetically and phylogenetically [7].

Indeed, the evidence that sleep serves essential functions is founded on several fundamental observations. First, sleep was spared by evolutionary pressures, indicating a crucial function, or functions, that cannot be easily circumvented [8]. Second, extended wakefulness beyond the normal sleep period necessarily leads to compensatory sleep rebound, underlying regulatory homeostatic mechanisms and the absolute need for a minimum amount of sleep [8]. Third, sleep loss has many harmful consequences for the organism, and, to date, investigations on the functions of sleep have mainly relied on studies of the consequences of sleep deprivation [8–10]. Finally, sleep is ubiquitous in the animal kingdom if one defines sleep to be simply a state of reduced activity, and/or reduced responsiveness to external stimuli [8]. However, in the absence of electrophysiological signs resembling those of mammals and birds, sleep in some animals such as reptiles, amphibians, fish and invertebrates may serve a function that is quite distinct from that served by human or mammalian sleep [8].

The noxious consequences of the lack of sleep have been known for a long time and have been used as a form of torture throughout history. However, the first reported experimental studies of sleep deprivation, especially the total absence of sleep, were published at the end the nineteenth century [11]. Conducted on dogs kept awake by constant activity or by using a bespoke cage keeping the animals awake without forced locomotion, they showed that total sleep loss led to ‘psychic exhaustion', severe brain degenerations and was lethal after 4–17 days. The second half of the nineteenth century also saw the emergence of clinical observations revealing the adverse effects of prolonged sleep deprivation and insomnia, provoking severe psychic disturbances such as delirium, hallucinations and emotional disruption [11,12]. Later studies on the effects of extended sleep loss in healthy people documented more detailed sleep-deprivation-induced psychopathological symptoms, including perceptual distortions, mood changes and psychosis [12].

These early studies shed light on the multiple deleterious effects of sleep loss, confirming the vital aspect of sleep and triggering the emergence of a growing interest in sleep in the scientific community. After the characterization of the homeostatic mechanisms underlying the stress response by Hans Selye in 1936 [13], some studies started to investigate the multiple facets of sleep, and in particular the consequences of sleep loss in the context of the so-called stress and coping processes [14,15].

## 3. Is sleep deprivation stressful?

Stress was initially defined as a ‘general adaptation syndrome' whereby an organism reacts to nocuous stimuli by non-specific physiological or behavioural responses [16]. The main neuroendocrine systems involved in the stress response are the autonomic sympatho-adrenal system and the hypothalamic–pituitary–adrenal (HPA) axis [17]. In this review, we only focus on the latter because it is responsible for the core hormonal response to homeostatic challenge [18], as well as it being, by far, the most studied aspect of sleep deprivation in the context of stress.

During a stressful situation, the brain of mammals responds by activating the HPA axis, which releases corticotropin-releasing hormone (CRH) from the hypothalamus. When CRH reaches the pituitary, the latter releases adrenocorticotropic hormone (ACTH), which triggers the secretion in the bloodstream of the steroid hormone glucocorticoids by the adrenal cortex (cortisol in primates and corticosterone in rodents) [18]. Glucocorticoids are essential for the adaptive response to stress, ultimately leading to the normalization of glucocorticoid release by the inhibition of the HPA axis activity through a negative feedback signal [18]. The basal activity of the HPA axis, and therefore glucocorticoid release, is largely orchestrated by the internal circadian clock located in the suprachiasmatic nucleus of the hypothalamus and exhibits a daily rhythm in both humans and rodents [19,20]. The secretion of glucocorticoid peaks just before the end of the resting period and decreases throughout the active phase until reaching a trough at the beginning of the sleeping period [20]. Interestingly, the exogenous administration of CRH induces an increase in wakefulness and a reduction in NREM sleep in humans [21] and rodents [22,23], and inhibits sleep-active neurons in the preoptic area of the hypothalamus [22]. The reduction or the increase of corticosterone also alter sleep architecture in rats [24], and human studies showed that sleep itself slightly dampens cortisol release whereas arousal is associated with cortisol burst [25,26]. However, while an increase in CRH suppresses the homeostatic response following sleep deprivation, the change of circulating corticosterone concentration does not seem to affect sleep rebound [24].

In this context, it is no wonder that alterations in glucocorticoid secretion following sleep deprivation or restriction have been documented [27]. However, in humans, moderate sleep loss has modest effects on HPA axis activity, which is affected differently among studies. For instance, chronic sleep restriction of 5 h or more per night for four to eight consecutive nights does not alter the cortisol release pattern, most probably because of endogenous compensatory mechanisms during sleep [28–31]. However, greater sleep loss characterized by a maximum of 4 h of sleep per night for one to six consecutive nights has been associated with dampened morning cortisol awakening response and increased afternoon/evening cortisol level [32–37], as well as reduced reactivity and slower recovery of the cortisol response [38]. Furthermore, a longitudinal study showed that recurrent short sleep is associated with an adverse cortisol secretion pattern [39]. The effect of sleep restriction on cortisol release seems to be dependent on when sleep loss occurs during the night and if subjects are usually awake or asleep at the time of sleep deprivation [40,41], highlighting the influence of complex interactions between sleep pressure and the subject's endogenous circadian and ultradian rhythms on HPA axis activity [42].

The flattening of the circadian release of cortisol has also been observed after one night of total sleep deprivation [43], despite most of the reports describing either an increase [33,44–47] or a decrease [48–52] in cortisol level following sleep loss. The same disparity is observed with sleep fragmentation and REM sleep deprivation [53,54]. These discrepancies could be explained by possible methodological limitations which might prevent an accurate assessment of the effect of sleep deprivation on cortisol release rhythmicity. However, and interestingly, some studies also failed to show a change in the HPA axis activity following total sleep deprivation when the experimental conditions were designed to avoid stress [55–57]. This shed light on the potential confounding effects of stress *per se* in sleep-deprivation experiments. Indeed, in addition to the immediate effects of sleep deprivation on the HPA axis activity, sleep loss has also been shown to affect subsequent stress reactivity. If one night of sleep deprivation did not increase responsiveness to an acute psychosocial challenge despite increased stress levels [58,59], poor sleep habits were associated with higher blood pressure and cortisol level during psychosocial stress [60]. Sleep quality seems to play a more important role in stress reactivity than sleep quantity [61].

The increase in HPA axis activity that is exhibited by some sleep-deprived individuals might be the direct consequence, at least in part, of the mental and physical load the subjects experience in order to stay awake rather than the effect of sleep loss alone. One could even consider sleep deprivation as a stressor *sensu stricto* (strictly speaking) in the event of a stress response beyond what is expected during relaxed wakefulness. This is no trivial matter considering the complex and reciprocal relationship between sleep and HPA axis activity [62,63], in addition to the well-established effects of stress on sleep homeostasis [64,65]. Whether the activation of the neuroendocrine stress system is the result of forced wakefulness or the stressful nature of the sleep-deprivation procedure is a fundamental question, and the absence of a clear answer has serious consequences for sleep research involving sleep deprivation. This is particularly true in animals, for whom sleep deprivation is necessarily achieved by subjecting them to potentially stressful movement-restricting novel environments or external stimulation [9]. Several sleep-deprivation protocols for rodents have been designed and used during the last 50 years, and understanding their specificities is crucial for the development of novel procedures to suppress sleep while minimizing, and even eliminating, any stress-associated confounding effects on the experimental outputs.

## 4. Do sleep-deprivation procedures in rodents affect the level of stress?

With the growth of sleep science from the 1970s and given the acknowledged, but poorly understood, bidirectional link between sleep and stress, some studies thus started to control for the stress-associated effects of their sleep-deprivation procedures in rodents as they became the model of choice for sleep studies [66,67]. Thereafter, the increasingly documented effect of sleep loss on the activity of the HPA axis, measured by the level of stress hormones, provided insight into the complex interrelated processes underlying sleep homeostasis and stress.

From the 1960s onward, several automated methods based on forced locomotion emerged in an attempt to standardize sleep-deprivation protocols. Treadmills or rotating wheels that can either move continuously or as soon as the animal displays behavioural and/or electrophysiological signs of sleep were used in rats [68,69]. However, after spending some time in the apparatus, the animals often find strategies to get some rest, such as running in the opposite direction to that of the treadmill or wheel and having short sleep episodes before waking up to avoid falling. If the amount of sleep suppression obviously depends on the movement speed imposed on the animals, it has been shown that an animal could sleep on average almost 40% of the time [70]. More importantly, major concerns have been raised about the confounding effects of exercise and fatigue on output measures [27], and particularly their associated stressful components. Using a rotating cylinder, an early study showed that 21.5 h of sleep deprivation in rats caused no significant increase in corticosterone compared with control animals with *ad libitum* (as much and as often as desired) sleep [71]. However, the release of corticosterone was greatly increased after 20 min of forced locomotion. Despite this study's conclusion stating that the overall low level of stress was unlikely to substantially alter sleep patterns during recovery, the immediate but transient effects of forced locomotion on corticosterone release, and thus subsequent effects on sleep parameters and underlying sleep-regulating mechanisms, cannot be ignored. Furthermore, other studies have shown that sleep deprivation, fragmentation or restriction by forced locomotion for 11–48 h in rats markedly increased the release of corticosterone [72–77]. In mice, 24 h of sleep deprivation using an activity wheel did not cause a significant increase in corticosterone [78], while another study showed that chronic sleep interruption during 14 days using a rotating drum induced an increase in corticosterone [79]. More recently, other paradigms have been developed in order to minimize the stress and/or physical fatigue of previous forced locomotion sleep-deprivation protocols. An apparatus consisting of two platforms alternatively moving below and above a water surface was designed to keep mice in constant motion without locomotion [80]. However, 10 h of total sleep deprivation using this novel paradigm did not prevent a significant rise of corticosterone [81]. Other methods, based on real-time EEG or EMG biofeedback systems triggering the rotation of cylindrical cages or running wheels when NREM sleep, REM sleep or inactivity are detected, were able to prevent sleep for 6–11 h in male rats and mice with a mild but non-significant increase in corticosterone [77,82].

The disc-over-water (DOW) apparatus was introduced in order to better control the influence of physical stimuli on experimental and control rats or mice [83–85]. In this paradigm, a sleep-deprived animal and its yoked control counterpart are housed on each side of a two-chambered cage separated by a central wall. The floor of the cage consists of a single flat disc, suspended over a tank of water, so that each animal lay on one half of the disc. When behavioural or electrophysiological signs of sleep are observed in the experimental individual, the disc rotates around the central axis of the apparatus and forces both animals to walk in the opposite direction in order to avoid falling into the water. This method ensures similar physical stimulation and locomotor activity for both experimental and control animals, while allowing the latter to sleep whenever the experimental one is awake and the disc immobile. However, the yoked control is inevitably partially sleep deprived when attempting to sleep at the same time as its experimental counterpart, with a respective reduction of total sleep time of around 30% and 90% [86]. The DOW paradigm is usually performed over an extended period of time, ranging from 2 days to one month. When not leading to the death of the experimental animals, the DOW applied for several days induced a severe stress syndrome including a decrease in body weight, enlarged adrenals and stomach ulcers, despite a reduction in corticosterone compared with baseline in male rats [85]. The decrease or the absence of alteration of the corticosterone level following sleep restriction by the DOW paradigm has also been observed in other studies in male rats [87–89]. However, male rats subjected to the DOW paradigm for days in order to achieve total sleep deprivation exhibited an increase in ACTH and corticosterone, while displaying only an increase in ACTH when deprived of REM sleep specifically [83]. In addition, one study showed that an increase in corticosterone level can be observed after 2 days of sleep deprivation in female rats compared with control animals that were not subjected to the DOW device [90]. Other protocols based on the same principles as the DOW, but without water, were also developed. In these apparatuses, experimental and yoked male mice subjected to a 9-day experiment displayed a significant increase in corticosterone only during the first day of sleep deprivation [91], while male rats sleep-deprived during 12 h by means of a gradual increase in the speed and the variability of the disc's rotation exhibited a slight increase in brain corticosterone concentration, but this did not exceed the normal circadian peak [92].

Other automated approaches involving tactile stimulations have been employed to fragment sleep in mice. A cage with a sweeping horizontal bar just above the floor was used to achieve 6 h of sleep fragmentation without altering corticosterone level [93], but later studies showed that the same procedure applied for 1–3 days induced an increase in corticosterone [94,95]. However, 3 days of sleep fragmentation using a rotating lever providing tactile stimulations did not seem to increase circulating corticosterone concentration in adolescent mice [96].

Nowadays, one of the most popular methods to achieve a total suppression of sleep in rodents is the ‘gentle handling' (GH) procedure, in which behavioural and/or electrophysiological signs of sleep are actively monitored by a trained experimenter who keeps the animal awake using gentle sensory stimulations [97–99]. When properly performed, the GH procedure can suppress almost all sleep activity [98]. The GH method has never been formally standardized across laboratories and encompasses a variety of different stimulations. Animals can be kept awake using external stimuli such as gentle tapping and/or shaking of the cage, mild noises, bedding disturbance or by touching the animal with a brush or directly with the hand [98,99]. Despite having been initially designed to minimize the spurious consequences of stress on sleep, the effects of GH on the HPA axis activity is more controversial than the consensually recognized stressful effects of sleep-deprivation protocols based on forced locomotion. The exposure to external stimuli between 2 and 24 h has been shown to induce an increase in corticosterone levels in male mice [100–105] and rats [106–110], as well as for very short periods of time in neonatal rats [110]. However, this increase was usually mild and within the range of normal amplitude observed during the undisturbed period. Standardizing GH protocols is not an easy task, as it is often necessary to adapt the amount of stimulation depending on the sleepiness and the drowsiness of each subject, which inevitably introduces variability. The number of stimulations applied to the animal typically increases throughout the sleep-deprivation procedure [103]. It has been shown that mice kept awake with as little disturbance as possible display lower corticosterone levels than animals kept awake using social stimuli or regular direct handling [111,112]. If rats seem to be less affected by stress during GH procedures [109,113], conflicting results have been reported regarding the habituation of mice to GH. Compared with undisturbed animals, 3 min of daily GH during 6 days in male mice induced no alteration in corticosterone release during the first day then an increase in corticosterone by the end of the sixth day [114], while the exact opposite was also described [115]. In addition, the familiarity an animal has with the experimenter can influence the consistency of the experimental results [116], while exposure to male experimenters is associated with a higher physiological stress response than exposure to female experimenters [117].

An alternative milder procedure to GH, aiming at inducing sleep deprivation by spontaneous exploratory and locomotor behaviour in rodents using novel objects or nesting material, has also been proposed [118–121]. Despite minimizing the number of external stimuli as well as allowing a more natural and spontaneous arousal state, the ‘novel objects' sleep-deprivation paradigm performed during 4 h in male rats has been shown to increase free corticosterone in hippocampal dialysates [122]. Furthermore, the presence of novel objects may be associated with learning effects [123]. Of note, the introduction of novel objects in the cage of sleep-deprived animals is most of the time used in combination with more classical GH techniques [101,113].

The inverted ‘flowerpot’ method was originally established in the 1960s to suppress REM sleep in cats [124] and was later adapted to rodents [125,126]. The experimental animal is placed on a small platform emerging from a water tank, while the control animal is usually placed on a larger platform. The size of the small platform allows the animal to squat and enter into NREM sleep. However, at the onset of REM sleep and associated muscle relaxation, the animal loses its balance and falls into the water, causing it to awaken. By contrast, the control individual can sleep *ad libitum* on the larger platform. The stressful aspects of this procedure have been raised in a number of studies, and an early report showed that male rats deprived of REM sleep (and food) for 24 h exhibited an increase in corticosterone and gastric ulceration [127]. Later studies also highlighted that selective REM-sleep deprivation for 1–5 days using the single platform method induces an increase in corticosterone and ACTH in male rats [128–135]. REM-sleep restriction for three consecutive days, 6 h daily, also induced an increase in corticosterone. Recently, a study showed that the administration of the single platform method on male mice for 1 day induced an increase in corticosterone, while 3 days of REM-sleep deprivation led to the death of some animals [94], further emphasizing the noxious aspects of the method. A modified version of the classic ‘flowerpot' method with multiple platforms in a larger tank, allowing the sleep deprivation of several animals at the same time, was later designed in order to remove stress-associated movement restriction and isolation [136]. An early study assessing adrenal hypertrophy, thymus atrophy, body weight loss and stomach ulceration induced by 3 days of sleep deprivation using the single and multiple platforms methods in male rats showed that, if both paradigms only caused mild stress compared with food deprivation, the multiple platforms protocol was less stressful than the classical platform technique [137]. However, another study also comparing the effects of the single and multiple platforms methods, applied during 4 days, on the HPA axis activity of male rats showed that both protocols caused an increased secretion of corticosterone and ACTH, and confirmed that this effect was stronger in the single platform paradigm [138]. The stressful aspect of the multiple platforms apparatus was further highlighted in several other studies showing that 18 h to 21 days of REM-sleep suppression induced an increase in corticosterone and/or ACTH in male rats [139–148] and mice [149,150]. Other related methods using two small platforms or a ‘grid over water' have been shown to also induce an increase in corticosterone in male mice after 2 and 3 days, respectively, [151,152]. In juvenile and male rats subjected to REM-sleep restriction for 14 or 18 h daily during a maximum of 21 consecutive days using the multiple platforms technique, a higher level of corticosterone has been observed than in non-sleep-restricted animals [153,154]. However, female rats that were REM sleep-restricted for 20 h daily for 6 days during pregnancy did not exhibit any increase in corticosterone release compared with control animals, while having higher relative adrenal weight [155]. The absence of any change in corticosterone release following REM-sleep deprivation for 2–3 days in the multiple platforms apparatus has also been observed in male and female rats [156,157].

One major limitation in most studies assessing the effect of forced wakefulness on stress through the activity of the HPA axis is that the level of stress-related hormones is usually measured at the end of the sleep-deprivation procedure. As several studies demonstrated that sleep deprivation using different paradigms can induce an early and transient increase in corticosterone before normalization [71,158–160], this is likely to mask any early increase in corticosterone that could potentially have an influence on later output measures.

## 5. What are the stress-associated consequences of sleep deprivation in rodents?

The activation of the HPA axis during sleep deprivation might have multiple physiological and behavioural effects. Whether these effects are the consequence of the increase in wakefulness or of the activity of the HPA axis is a question for which there is no simple answer.

For instance, the regulation of adult hippocampal neurogenesis has been linked to both stress and sleep. Several animal studies highlighted a negative effect of sleep loss on hippocampal cell proliferation and neurogenesis [134,144,150,161], while others reported a positive effect after short periods of sleep deprivation [162,163]. The implication of the HPA axis in the alteration of hippocampal neuronal plasticity following sleep deprivation has also been debated. A study in male rats subjected to REM-sleep deprivation reported that the reduction in cell proliferation and neurogenesis in the hippocampus was abolished by the suppression of the corticosterone surge [134]. However, other studies showed that the inhibitory effect of sleep fragmentation and REM-sleep deprivation on hippocampal cell proliferation and neurogenesis of male mice was not dependent on the increased level of corticosterone induced by sleep loss [144,150,161]. Nevertheless, if a sleep-associated rise in corticosterone has negligible impacts on hippocampal neuroplasticity, an unwanted stress-associated corticosterone surge accompanying sleep-deprivation protocols might have a greater effect.

As in humans, sleep deprivation or fragmentation in rats alters stress reactivity [75,76,164], but also anxiety [74] and despair behaviour [89]. Similarly, specific REM-sleep deprivation facilitates a subsequent corticosterone response to a mild stressor [138], while chronic stress during the REM-sleep suppression procedure increases REM-sleep rebound [130]. Moreover, studies reported that pharmacological inhibition of corticosterone synthesis during REM-sleep deprivation in male rats resulted in impairment of sleep rebound [135], suggesting that the activation of the HPA axis following REM-sleep suppression is necessary for proper sleep recovery. The link between REM sleep and stress-associated behaviour has been further highlighted by studies reporting that, in addition to the increased corticosterone level, specific REM-sleep deprivation induces higher anxiety and depressive-like behaviour in rats [145,146,153,154,165]. All these findings underline the crucial effects of sleep loss, and in particular the suppression of REM sleep, on physiological and behavioural stress coping mechanisms.

## 6. Can we overcome undesired stress-related consequences of sleep-deprivation procedures in rodents?

Given the intricately linked mechanisms between sleep loss and stress, separating the consequences of the sleep-deprivation procedure in animals from the stressful protocol-related external stimulations is challenging. As pointed out by a myriad of sleep-deprivation studies measuring the activity of the HPA axis, extended wakefulness is often associated with a certain degree of stress. Undoubtedly, this can confuse the conclusions drawn from all research involving sleep-deprivation procedures. This could be prevented by minimizing or avoiding human intervention and sensory–motor stimulation in order to limit any stress-associated confounding factors. The technological progress seen in recent years might offer interesting alternative solutions to the classical sleep-deprivation paradigms and their stress-inducing components.

In particular, the novel developments of gene-engineering techniques and biotechnological tools, such as chemogenetics and optogenetics, provide the possibility to activate or inhibit neurons in a time-, type- and region-specific manner [166]. After light-sensitive ion channels, or designer receptors exclusively activated by designer drugs, are expressed in targeted cells, optogenetics allows neuronal depolarization or hyperpolarization with pulses of light while chemogenetics provides the ability to modulate neuronal firing for several hours with the single administration of a designer drug. With the advancements in mapping brain sleep circuitry [1], ‘genetic sleep deprivation' can now be accomplished without stress-associated sensory stimulation via the inhibition of sleep-promoting neurons or the activation of wake-promoting neurons [7]. This could be done by directly targeting sleep- or wake-active neurons, or through the manipulation of neuronal populations that stimulate wake-promoting, or inhibit sleep-inducing, neurons. For instance, lesion of the ventrolateral preoptic (VLPO) area of the hypothalamus, one of the first sleep-controlling brain regions discovered [167,168], reduces sleep by approximately 50% without inducing hyperarousal or obvious signs of stress in male rats [169,170]. Recently, it was found that sleep-active neurons in the preoptic area are spatially intermingled with wake-active neurons [171]. In male mice, the optogenetic activation of neurons located in the preoptic area and expressing *γ*-aminobutyric acid (GABA), the main inhibitory neurotransmitter of the central nervous system, induces a strong increase in wakefulness [171]. A similar effect is achieved by silencing of the GABAergic neurons of the preoptic area that specifically project into the tuberomammillary nucleus [171]. Moreover, the photoinhibition of galanin-secreting neurons in the VLPO decreases NREM sleep by 60% [172]. In male mice, sleep-promoting neurons in the VLPO can also be inhibited indirectly via the chemogenetic activation of VLPO-projecting GABAergic neurons in the lateral hypothalamus [173]. Furthermore, the activation of GABAergic neurons in other structures in the basal forebrain also promotes wakefulness [174].

However, targeting a single sleep-active neuronal cluster might not be the most efficient way to suppress sleep and could be more adapted for sleep fragmentation protocols [7]. Thus, the direct or indirect activation of brain circuits that promote arousal may seem a better strategy for total sleep deprivation. Indeed, repeated chemogenetic activation of the pontine parabrachial nucleus of male rats has been found to potently increase wakefulness over 4 days [175], while acute chemogenetic activation of glutamatergic neurons in the ventral tegmental area completely suppresses sleep for 5 h in male mice [176]. Furthermore, the chemogenetic activation of glutamate-releasing neurons of the supramammillary region in male mice strongly promotes wakefulness for up to 12 h [177] and has successfully been used to carry out a 9 h sleep-deprivation protocol [178].

Beyond the possibilities of suppressing sleep by activating or inhibiting neuronal populations without sensory–motor stimulations, sleep-deprivation procedures can also benefit from sleep detection algorithms. Traditionally, the classification of the vigilance states is performed by trained human experts via visual inspection of the EEG features. While visual EEG classification is still considered the gold standard for sleep scoring, the considerable progress in computational technologies has made possible the improvement of automated real-time sleep scoring algorithms. Recently, novel paradigms for REM-sleep deprivation aiming at reducing human intervention and locomotion-related fatigue were developed. Using real-time automated sleep stage recognition by online analysis of EEG spectral components [179] or more efficiently by using an unsupervised machine learning algorithm [180], REM-sleep restriction was, respectively, achieved via gentle cage vibration in male mice or sudden shake of the cage floor in male rats. Although these studies did not assess the effect of REM sleep loss on HPA axis activity, one report using total sleep deprivation through delivery of air puffs when the EEG and EMG characteristics of sleep are automatically detected did not induce an increase in corticosterone or ACTH in male rats [181].

Genetic sleep deprivation is likely to reduce sleep-deprivation-related stress by suppressing the need for human intervention and sensory–motor stimulation that are required in conventional sleep-deprivation protocols. Moreover, this approach allows the animal to behave naturally in its home cage or any other familiar environment. Activating or inactivating specific neuronal populations in order to prevent sleep is not inconsequential though, because it could also trigger undesirable neurophysiological epiphenomena in brain regions that are not involved in sleep regulation and then potentially interfere with the primary outputs of the experiments. In addition to unwanted physiological and behavioural responses, this might also influence the HPA axis considering its broad and complex neuronal circuitry [18]. Careful consideration should therefore be given to selecting the most appropriate neurons in the most relevant location in order to minimize adverse effects. In addition, the latter could be avoided by using more subtle approaches, such as real-time analysis of EEG and EMG signals triggering the indirect optogenetic activation or inactivation of sleep-suppressing or sleep-promoting brain nucleus in order to avoid continuous stimulation.

## 7. Conclusion

Despite extensive research since the discovery of the fundamental importance of sleep, its functions and functioning are still a matter of debate. However, a consensus exists regarding the cardinal contributions of sleep to mental and physical health with a multitude of studies pointing out the adverse consequences of disturbed sleep [182–185]. The high prevalence of insomnia and poor sleep quality in modern societies highlights the need for further and better research aiming at unveiling the mechanisms behind sleep regulation and function [186]. To that end, the study of the consequences of sleep loss are crucial, yet suffer from inherent confounding factors that are difficult to bypass. As argued in this review, the main bias that is likely to affect the results of sleep-deprivation procedures is undoubtedly stress. This can be particularly problematic for preclinical studies investigating sleep mechanisms in stress-related diseases such as mood and neurodegenerative disorders [182,183]. The use of more sophisticated sleep-deprivation methods involving the control of genetically defined neurons through optogenetic or chemogenetic methods, associated or not with online interpretation of brain electric signals, could be a leap forward to address the aforementioned issues. However, the technical challenges and financial considerations of genetic sleep deprivation still prevent it from taking precedence over more classical sleep-deprivation procedures. Meanwhile, given the specificities of each sleep-deprivation procedure in terms of confounding effects, the selection of a sleep-deprivation protocol should be done in order to minimize the influence of the procedure *per se* on the primary output measures. More importantly, for the sake of the experimental results as well as the animals themselves, no sleep-deprivation experiment should be carried out without ascertaining that the experimental conditions are as stress free as possible.

## Data accessibility

This article has no additional data.
